# Novel micropatterning technique reveals dependence of cell-substrate adhesion and migration of social amoebas on parental strain, development, and fluorescent markers

**DOI:** 10.1371/journal.pone.0236171

**Published:** 2020-07-23

**Authors:** Richa Karmakar, Christoph Schich, Nadine Kamprad, Vanessa Scheller, Edgar Gutierrez, Alex Groisman, Wouter-Jan Rappel, Marco Tarantola

**Affiliations:** 1 Department of Physics, University of California, San Diego, La Jolla, California, United States of America; 2 Institute for Dynamics of Complex Systems, Goettingen, Germany; 3 Max Planck Institute for Dynamics and Self-Organization, Göttingen, Germany; Université de Genève, SWITZERLAND

## Abstract

Cell-substrate adhesion of the social amoeba *Dictyostelium* *discoideum*, a model organism often used for the study of chemotaxis, is non-specific and does not involve focal adhesion complexes. Therefore, micropatterned substrates where adherent *Dictyostelium* cells are constrained to designated microscopic regions are difficult to make. Here we present a micropatterning technique for *Dictyostelium* cells that relies on coating the substrate with an ∼1*μ*m thick layer of polyethylene glycol (PEG) gel. We show that, when plated on a substrate with narrow parallel stripes of PEG-gel and glass, *Dictyostelium* cells nearly exclusive adhere to and migrate along the glass stripes, thus providing a model system to study one-dimensional migration of amoeboid cells. Surprisingly, we find substantial differences in the adhesion to PEG-gel and glass stripes between vegetative and developed cells and between two different axenic laboratory strains of *Dictyostelium*, AX2 and AX4. Even more surprisingly, we find that the distribution of *Dictyostelium* cells between PEG-gel and glass stripes is significantly affected by the expression of several fluorescent protein markers of the cytoskeleton. We carry out atomic force microscopy based single cell force spectroscopy measurements that confirm that the force of adhesion to PEG-gel substrate can be significantly different between vegetative and developed cells, AX2 and AX4 cells, and cells with and without fluorescent markers. Thus, the choice of parental background, the degree of development, and the expression of fluorescent protein markers can all have a profound effect on cell-substrate adhesion and should be considered when comparing migration of cells and when designing micropatterned substrates.

## Introduction

Cell migration plays an essential role in many biological processes, including wound healing, development, chemotaxis and cancer metastasis [1–4]. Traction is required for cell migration and can be provided by cell-cell adhesion or by adhesion to the extracellular matrix (ECM). Mammalian cells have dedicated adhesion complexes, consisting of various specialized molecules including integrins [5, 6]. These complexes bind to the ECM and to facilitate adhesion and migration in mammalian cell cultures, substrates are often coated with cognate ECM proteins. It is therefore relatively easy to confine the adhesion of mammalian cells to specific regions of a substrate by selectively coating these regions with ECM and applying an adhesion blocking treatment to the rest of the substrate.

A widely used type of such blocking treatment is the coating of the substrate with a macromolecular “brush” of polyethylene glycol (PEG) [7–9]. A micropattern of adhesive islands or stripes on an otherwise adhesion-blocked surface can be created using microstamping or selective exposure to UV-light using, e.g., a photomask or laser scanning [10–14]. These micropatterned substrates can then be used to study the effect of the shape and size of the region available for cell adhesion on cell phenotype and migration [15–17]. In particular, when the region available for cell attachment is a narrow stripe, cell migration becomes nearly one-dimensional (1D), often exhibiting morphodynamics and modes of locomotion that are distinct from those on a 2D substrate and similar to those of cells migrating in 3D ECM [10, 16].

Due to its relative ease of handling and genetic modification, the social amoeba *Dictyostelium discoideum* is often used to study migration and chemotaxis (motion guided by chemical gradients) [18, 19]. Cell motion in *Dictyostelium* cells arises from the formation of actin-filled protrusions (pseudopods) at the front and myosin-based retraction at the back of the cell [20]. Importantly, *Dictyostelium* cells do not make integrin mediated focal adhesions and the required adhesive forces between the cell membrane and the substrate are thought to involve innate van der Waals forces as well as electrostatic and hydrophobic contributions [21].

*Dictyostelium* cells are inherently “sticky” and have the ability to adhere to and migrate on a wide variety of surfaces. [21–23]. Therefore, the task of creating micropatterned surfaces that restrict cell adhesion and migration to specific regions of the substrate is different and more challenging for *Dictyostelium* cell than for most adherent mammalian cells. Designing these micropatterns can be desirable if one wants to investigate the effect of confinement on the migration of *Dictyostelium* cells. Furthermore, a substrate with a micropattern of narrow stripes, rendering *Dictyostelium* cell migration effectively 1D, would make it easier to analyze and model the migration, potentially helping better understand cell migration.

It has been reported that the adhesion of *Dictyostelium* cells to a glass substrate can be substantially reduced if the substrate is coated with Pluronic, a block copolymer of PEG and polypropylene oxide [24]. When plated on a substrate with a lithographically generated periodic micropattern of 10 *μ*m wide stripes of untreated glass and Pluronic-treated glass, the majority of cells were shown to adhere to glass stripes. Nevertheless, the protocol used to create the micropattern was complex and included, among other steps, the spin-coating of the glass substrate with a photoresist, exposing it to UV light through a photomask, and removing the photoresist with a special developer. In addition, as much as 15% of cells still adhered to PEG-coated stripes, and no data were provided on, whether *Dictyostelium* cells were able to migrate on this micropatterned substrate and whether their migration was limited to stripes without PEG. It has also been reported that *Dictyostelium* cells do not adhere to commercial glass substrates with high densities of PEG molecules [25]. However, no micropatterned substrates with this type of PEG coating are currently commercially available.

In this report, we present an experimental protocol for creating a micropattern of narrow stripes of ∼1 *μ*m thick PEG-gel on a standard microscope coverglass by filling a microfluidic perfusion device with a PEG-gel pre-polymer with a near-UV photo-initiator and exposing it to UV-light derived from an LED. We test the ability of this micropattern to selectively block adhesion using starved (developed) and non-starved (vegetative) cells, from the axenic strains AX2 and AX4, which are derived from the same isolate (NC4) [26, 27]. In addition, we test the substrate adhesion of cells that express fluorescent protein markers. These markers are a powerful experimental tool, making it possible to obtain information on localization and activity of proteins in live cells in real time and on a sub-cellular level. Since actin polymerization at the cell front and myosin-mediated retraction at the cell rear are major components in cell migration, we focused on fluorescent proteins genetically fused to actin and myosin.

We find that the PEG-gel coating prevents the adhesion of developed *Dictyostelium* cells of the axenic strain AX4, constraining their migration to ∼10 *μ*m wide stripes of plain glass between the PEG-gel stripes, thus, providing a model for studying 1D migration of *Dictyostelium* cells. Surprisingly, however, we find that developed *Dictyostelium* cells of the axenic strain AX2 are able to adhere to PEG-gel surface. Furthermore, we find that developed AX2 cells expressing fluorescent proteins that are genetically fused to the cytoskeletal proteins actin and myosin, are unable to adhere to the PEG-gel surface and that, just as with the AX4 cells, their migration on the micropatterned substrate is constrained to glass stripes. Finally, AX2 cells, not expressing any fluorescent proteins but in a vegetative rather than developed state, show significantly preferred adhesion to glass vs. PEG-gel surface, making them similar to developed AX4 cells.

To better understand the adhesion of *Dictyostelium* cells and PEG-gel and glass surfaces, we measured the forces between cells and substrate using Single Cell Force Spectroscopy (SCFS). SCFS is an Atomic Force Microscopy (AFM)-based technique in which cells are repeatedly brought into contact with a substrate and pulled away from it and which can determine maximum forces and total work of adhesion [22, 28]. From experiments with developed AX4 and AX2 cells, we find that the difference between the maximum force on PEG-gel and glass surfaces is significant for AX4 cells but not significant for AX2 cells, consistent with our observations on adhesion and migration on micropatterned substrates. When developed AX2 cells express cytoskeletal fluorescent protein markers for actin or myosin their force of adhesion to glass becomes significantly greater than to PEG-gel, also consistent with our experiments on micropatterned substrates. Finally, our SCFS experiments show that vegetative AX2 cells adhere significantly stronger to glass than to PEG-gel, again in agreement with our experiments on micropatterned substrates.

Our study shows how to create micropatterned substrates with a high degree of adhesion specificity. Furthermore, our results show that, along with the developmental state of the cell (vegetative vs. developed), the choice of a specific axenic strain and the expression of fluorescent protein markers can have a profound effect on cell-substrate adhesion and should be considered when comparing adhesion and migration of cells.

## Materials and methods

### Cell culture and preparation

In this study, we used two axenic *Dictyostelium* strains AX2 and AX4. In addition, wild type (WT) AX4 cells were transformed with a construct in which the regulatory region of actin 15 drives genes encoding a fusion of GFP to LimE (Δ coil LimE-GFP) and a gene encoding a fusion of RFP to coronin (LimE-GFP/corA-RFP) [29]. In addition, we used AX4 cells with GFP tagged LimE (Δ coil LimE-GFP). We used the following single transformations for WT AX2 cells: GFP tagged MyoII (MyoII-GFP), GFP tagged LimE (Δ coil LimE-GFP), and GFP tagged alpha-tubulin (alpha-tubulin-GFP). We also used the double transformation GFP tagged MyoII and RFP tagged LimE. Cells were grown on a shaker in HL5 medium which contained 35.5g HL5 media (^®^ FORMEDIUM)/L of DI water [30] in shaking condition. When cells reached a density of 1-2 × 10^6^ cells/mL, they were harvested by centrifugation, washed in *KN*_2_/Ca buffer (14.6 mM *KH*_2_ *PO*_4_, 5.4 mM *Na*_2_ *HPO*_4_, 100 *μ*M *CaCl*_2_, pH 6.4), and re-suspended in *KN*_2_/Ca at 10^7^ cells/mL. These cells were used as vegetative cells. For developed cells, we kept the vegetative cells on a shaker with pulses of 50nM cAMP added every 6 minutes for 5 hrs.

### Preparation of uniformly PEG-gel coated substrates for micropipette and SCFS experiments

To prepare a substrate uniformly coated with PEG-gel, we carried out the following steps. First, we cleaned a #1.5, 47 mm diameter microscope coverglass with water and ethanol and blow-dried it. Then, we oxygen plasma-treated the glass surface for 10 s and exposed it to the vapor of 3-(Trimethoxysilyl) propyl Methacrylate (Aldrich^®^) at 77°C for 30 mins. A 30% Polyethylene Glycol Diacrylate (PEG-DA) pre-polymer solution was prepared by mixing PEG-DA (with average M_n_ = 900, Aldrich^®^) with a 0.03% aqueous solution of VA086 in a 3:7 ratio by volume. A ∼100*μ*L drop of the solution was dispensed onto the center of the cover glass and squeezed to a thin layer by placing an untreated #1.5, 30 mm diameter round cover glass on top, gently pushing this round cover glass with a pipette tip, and removing the excess solution. VA086 is a near-UV photo-initiator that cross-links PEG-DA molecules (thus, converting the PEG-DA solution into a PEG-gel) by binding to the acrylate groups and also links PEG-DA chains to the acrylate groups on the glass surface. Cross-linking of the pre-polymer solution was accomplished by purging O_2_ in an N_2_ chamber and exposure for 60s to UV light from a home-built 365 nm LED light source with an intensity of ∼360 mW/cm^2^ (total exposure of 2.2 J/cm^2^). After the top round cover glass was removed, the bottom cover glass had a thin layer of PEG-gel covalently bonded to the glass surface. The thickness of this layer was measured by depositing fluorescent beads on both the glass and PEG surface and measuring the distance along the z-axis between the beads on the two surfaces using confocal microscopy. This measurement revealed a PEG gel thickness of ∼4 *μ*m.

### Preparation of micropatterned PEG-gel substrates

**Fig 1:**
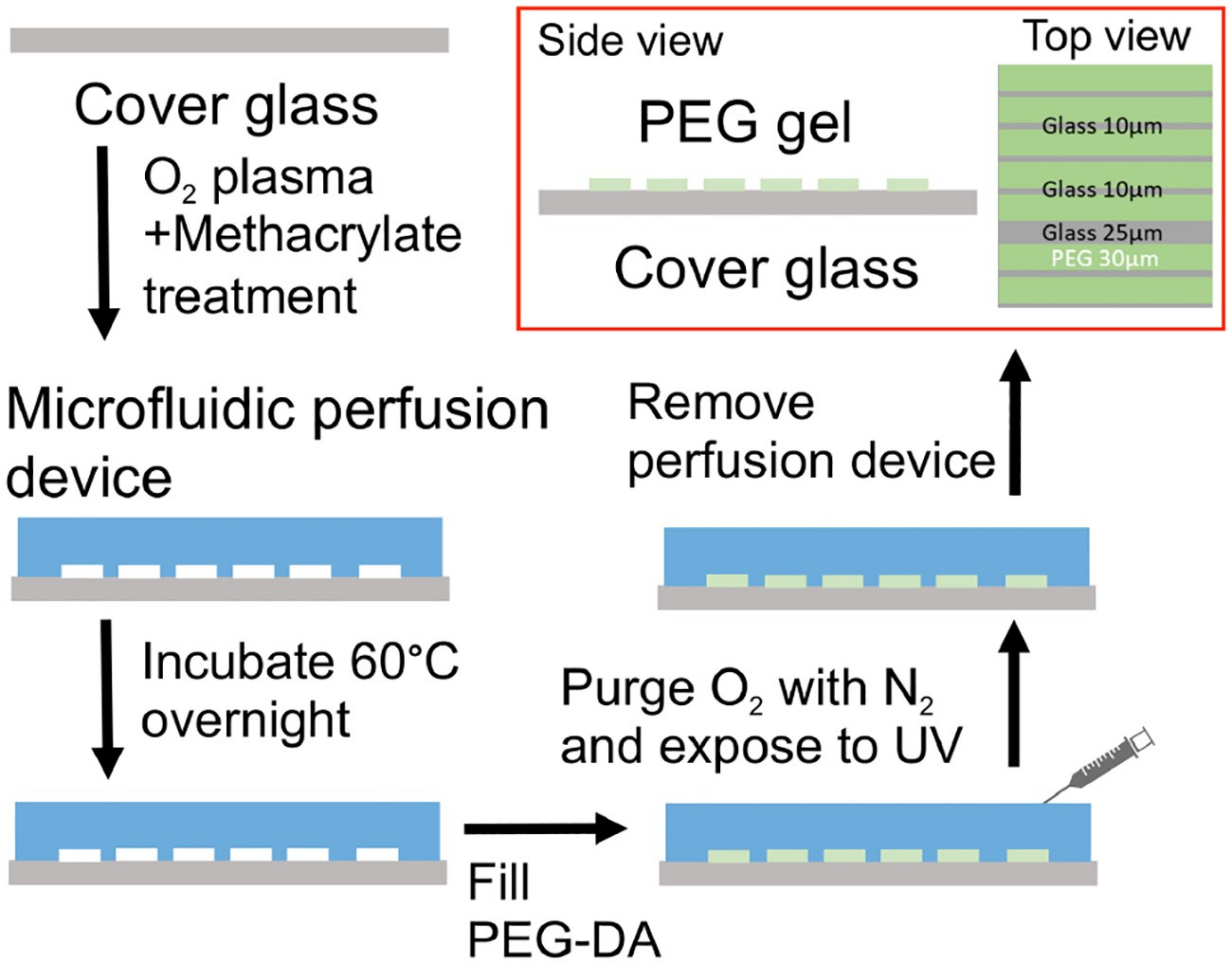
Schematic steps in micropattern substrate fabrication process. A micropattern of alternating PEG-gel and glass stripes is created by attaching a microfluidic chip to a clean microscope coverglass, reversibly bonding the device to the coverglass by overnight incubation at 60°C, filling microchannels of the devices with PEG-gel pre-polymer, purging O_2_ in an N_2_ chamber, cross-linking the PEG-gel by a UV-exposure, and detaching the microfluidic chip from the coverglass.

Our protocol for the preparation of micropatterned substrates is schematically shown in Fig 1. Micropatterns of PEG-gel were produced on #2, 50x35 mm microscope coverglasses and had periodic arrays of stripes, with each repeating unit of the array containing one ∼25*μ*m wide and four ∼10*μ*m wide glass stripes with ∼30*μ*m wide stripes of ∼1.5*μ*m thick PEG-gel between the glass stripes. The treatment of the glass surface and preparation the PEG-gel pre-polymer were the same as for the uniformly coated substrates described above. Therefore, in these substrates, the surface of glass was always functionalized with methacrylate. In the remainder of this manuscript, we refer to this functionalized glass simply as glass. The micropatterns were generated using a microfluidic PDMS chip that had a periodic array of ∼1.5*μ*m deep, ∼30*μ*m wide microchannels with one ∼25*μ*m wide and four ∼10*μ*m wide partitions between the microchannels in each repeating unit. As with the uniformly coated substrate, to determine the height of the PEG-gel stripes, we attached fluorescent beads to the surface of the PDMS chip that was used to generate the micropattern of PEG-gel stripes and imaged the beads with confocal microscopy. The microfluidic chip was attached to the coverglass, the microchannels were filled with the PEG-gel pre-polymer, and the assembly was placed into an N_2_ filled chamber for approximately 1 hr (to remove oxygen from the PDMS chip and PEG-gel pre-polymer) and exposed to the total of 2.2 J/cm2 of 365 nm UV-light to cross-link PEG-gel, as described above. After the UV exposure, the PDMS chip was removed, leaving behind PEG-gel stripes in places where microchannels used to be and glass in places where the PDMS partitions used to be.

### Rigidity measurement of the PEG gel

To assess the elastic modulus of the 30% PEG gel that we made with the 900 Da PEG molecules, we prepared a 4 mm thick layer of the gel and measured its rigidity with a durometer. The Shore A rigidity, *S*, was found to be *S*∼60. The elastic modulus, *E*, was estimated using the equation *E* = (0.0981(56 + 7.72*S*))/(0.138(254 − 2.54*S*)) [31] and was found to be E∼3.6 MPa. This value of *E* is well above the recently reported experimental range of substrate rigidity sensing for *Dictyostelium* cells (0.1-0.5 kPa) [32]. Therefore, the reduced rigidity of the PEG-gel stripes as compared with glass stripes is not expected to have any effect since *Dictyostelium* cells perceive both substrates as infinitely rigid.

### Adhesion experiments on uniform and micropatterned substrates

Cells were plated on glass surfaces, uniform PEG-gel surfaces, or micropatterned substrates and allowed to settle and attach for 10 min. On the uniform substrates, we used a micropipette filled with the chemoattractant cAMP to determine the cells’ ability to chemotax. For the micropatterned substrates, we imaged the cells with a 10x objective and experiments were repeated at least 4 times. We computed the total area of the two different surfaces and, using ImageJ, we determined the location of cells relative to these two surfaces. Specifically, we applied a threshold to each image and, following the filling of holes, watershed segmentation, and removal of outliers, created a binary image. The total number of cells was then counted by the ImageJ module “Analyze Particles”, and the number of cells on the PEG-gel stripes was determined manually. For comparison and error estimation, we also used a manual count without any image processing to estimate that the uncertainty in detection of the number of cells on each surface is less than 3%. We then calculated the density of cells on glass and PEG-gel surfaces, *ρ*_*glass*_ and *ρ*_*PEG*_, by dividing the number of cells by the respective surface areas. As a quantification of the distribution of cells on the glass and PEG-gel, we report here their relative coverage. This is computed by taking the ratio of this density (*ρ*_*glass*_ or *ρ*_*PEG*_) and the sum of densities (*ρ*_*PEG*_ + *ρ*_*glass*_) and expressing it in terms of a percentage value. In a control experiment, designed to rule out topographic guidance, we plated cells onto a flat untreated PDMS substrate with 1.5 *μ*m deep grooves. The topography of this substrate was a replica of the topography of the micropatterned PEG-gel substrates.

### Single cell force spectroscopy (SCFS)

SCFS experiments were carried out as described previously [28] and shown schematically in S1 Fig. Briefly, substrate adhesion was analyzed by an SCFS capable atomic force microscope (AFM, Asylum MPF-3D Bio, Asylum Research, UK, equipped with an unusual high z-range piezo allowing the usage of 30 *μ*m to detach specimen from more adhesive surfaces, while still containing xz scanners for imaging and movement purposes as well) mounted on an Olympus microscope (IX71, with 40x objectives) and a CCD Camera (ANDOR Zyla 4.2 sCMOS) for optical control of adhesion. Tipless cantilevers were used (Arrow TL2-50, Nano World, Switzerland) with a mean spring constant of *k* = 0.03 Nm^−1^, calibrated with the thermal noise method [33] before attaching the first cell. The adhesion between cell and cantilever was increased based on preoptimized protocols [22] using a commercially available polyphenolic adhesive protein mixture (Corning^®^ Cell-Tak™, BD Bioscience, USA).

Clean microscope coverglass plates (35mm diameter and 1mm thickness) were provided by the AFM manufacturer to match the biochamber geometry (111.425, Asylum Research, Santa Barbara). Half the coverglass was coated with uniform PEG-gel as described above while the other half remained uncoated and thus consisted of glass that was functionalized with methacrylate. Cells were developed as detailed above and transfered to phosphate buffer. Cells were plated on the coverglass and allowed to settle and attach for 10 min. To avoid using cells that have adapted their adhesion to continuous PEG-gel exposure, we only used cells from the untreated (glass) part of the coverglass. Furthermore, only cells that exhibited active morphological changes and protrusive activity were used in the experiments. Single cells were allowed to establish adhesion to the cantilever during a time period of 2 min under optical control. Once a cell adhered to the tip, cycles of approachment and retraction towards the PEG-gel substrate were initiated (S1 Fig). We used SCFS parameters that were previously optimized for *Dictyostelium* cells: an approachment/retraction velocity of 2.5*μ*m/s, a contact force of 500*p*N and a contact time of 30*s* [28]. After a relaxation time of 30s the cycle is repeated with the same parameter set between 5 and 10 times. The resulting force distance (FD) curves were analyzed with a customized Matlab script which determined the minimum of the FD curve, representing the maximum adhesion force F_max_, and the integral between the FD curve and the baseline, representing the adhesion work W_Adh_ (S1 Fig).

### Statistics and reproducibility

P values were computed with the Wilcoxon rank sum test using IgorPro (version 8.01, wavemetrics, Lake Oswego, OR 97035 USA). The significance threshold was set at a p-value of 0.05 with *** corresponding to p<0.001, ** to p<0.01, and * to p<0.05. Values are reported as median (interquartile 1- interquartile 3) or as mean ± standard deviation.

## Results

### Adhesion and migration on uniformly coated substrates

#### Developed cells

**Fig 2:**
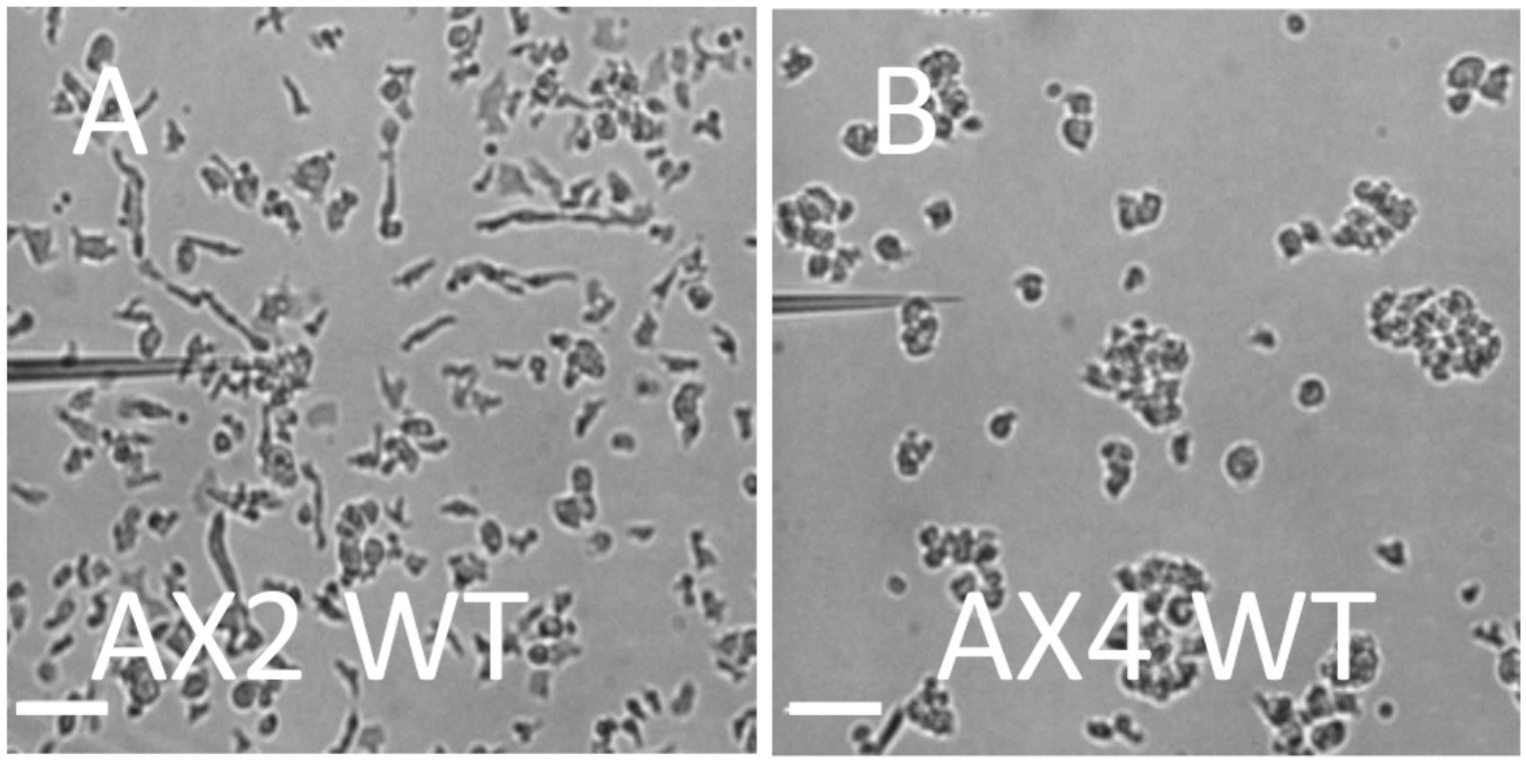
Developed WT cells on a uniform PEG-gel surface. Micrographs of AX2 (A) and AX4 (B) exposed to a gradient of chemoattractant cAMP leaking from a pipette. AX2 cells adhere to the surface and migrate toward the leaky pipette, while the AX4 cells remain in clumps and do not migrate (Scale bar: 50 *μm*).

We first examined the migration of developed WT AX2 and AX4 cells on substrates that were uniformly coated with PEG-gel (see Methods). To this end, we plated cells onto the substrate and determined their ability to migrate towards a micropipette leaking the chemoattractant cAMP. When first settled onto the substrate, both AX2 and AX4 were present in clumps. The AX2 clumps were relatively small and, after a short while, AX2 cells adhered to the surface and started to move, breaking the clumps. Importantly, the resulting single cells moved towards the pipette, demonstrating that WT AX2 cells can gain the traction required for motion on PEG-gel surfaces (Fig 2A and S1 Video). The speed of migration was measured as 14±3*μm*/*min* (N = 10), comparable to the speed of AX2 cells migrating on glass.

When plated on PEG-gel, AX4 cells behaved very differently from AX2 cells. In suspension, AX4 cells form larger clumps than AX2 cells, presumably due to greater cell-cell adhesion. These clumps remained largely unchanged even after 12 min in a gradient of chemoattractant (S2 Video) and AX4 cells did not migrate towards a pipette with cAMP (Fig 2B). Thus, unlike AX2 cells, WT AX4 cells are unable to gain sufficient traction for migration on the PEG-gel surface.

#### Vegetative cells

The behavior of vegetative AX2 cells plated on a PEG-gel surface was largely similar to the behavior of developed AX2 cells. They became elongated and started moving randomly, apparently gaining enough traction for migration (S3 Video). Vegetative AX4 cells plated on a PEG-gel surface had less elongated shapes and displayed less migration than AX2 cells (S4 Video). Nevertheless, unlike developed AX4 cells, vegetative AX4 cells were able to adhere to PEG-gel surface and gained sufficient traction to migrate on it.

### Adhesion and migration on micropatterned substrates

#### Developed cells

**Fig 3:**
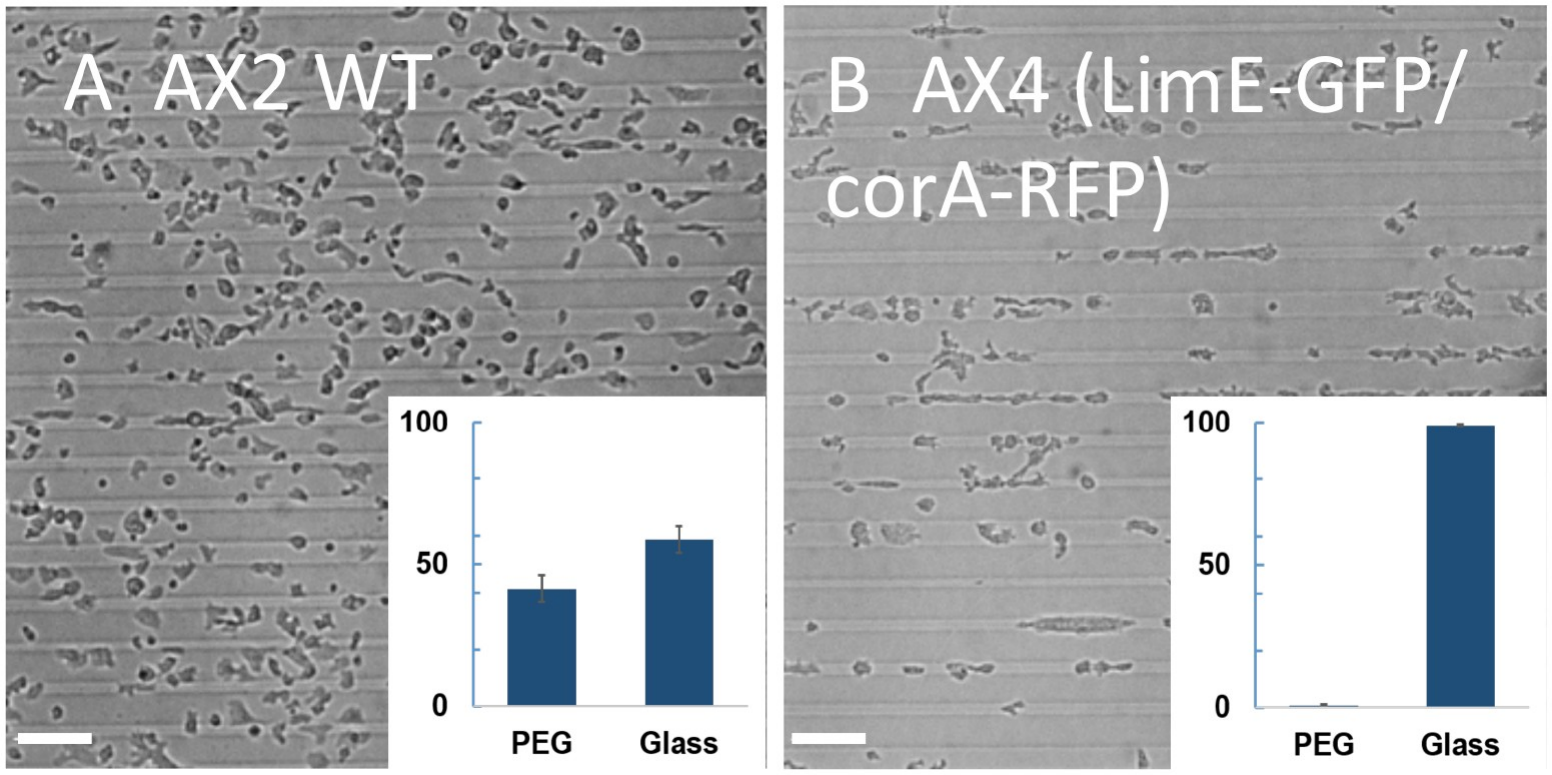
Developed cells on micropatterned substrates. Micrographs of WT AX2 cells (A) and AX4 cells expressing LimE-GFP and Coronin-RFP (B), 10 min after plating. Insets: relative coverage of cells on PEG-gel and glass stripes (expressed here and elsewhere as a percentage). Whereas AX2 cells adhere to PEG-gel and glass in comparable proportions, AX4 cells are found nearly exclusively on glass stripes. (Scale bar: 50 *μm*).

To investigate the ability of developed *Dictyostelium* cells to adhere to micropatterned surfaces, we designed a pattern which consisted of an array of stripes, with one ∼25*μ*m wide and four ∼10*μ*m wide stripes of glass and with ∼30*μ*m wide stripes of PEG-gel in between each repeating unit of the array (see Methods and Fig 1). When plated on these micropatterned substrates, WT AX2 cells spread out and broke up the clumps they formed when first settled onto the substrate. Consistent with our results with uniform PEG-gel (Fig 2A), cells were able to adhere to both the PEG-gel and glass surface (Fig 3A). This is quantified in the insert of Fig 3A where we report the number of cells on the glass and the PEG-gel surface. This quantification shows that cells adhered to PEG-gel and glass surfaces in almost equal numbers (41±5% vs. 59±5%; N = 2107).

When WT AX4 cells were plated on the micropatterned substrate, many of the large clumps that cells formed when settled on the substrate (Fig 2B) remained largely intact over extended time intervals, suggesting that the stripes of PEG-gel prevent cells in these clusters from adhering to the substrate. As a result, it was very difficult to study the migration of WT AX4 cells on the micropatterned substrate and to quantify the relative coverage of cells on glass vs. PEG-gel stripes. Looking for AX4 cells that would be able to adhere to the micropatterned substrate, we tested AX4 cells expressing fluorescent protein markers for LimE and coronin (see Methods), and found them forming substantially smaller clumps than WT AX4 cells. After a short while on the substrate, these relatively small clumps broke up into single cells, indicating that these fluorescent AX4 cells are able to adhere to the micropatterned substrate and migrate on it (Fig 3B). A quantification of the percentage of cells located on the glass stripes and on the PEG-gel stripes is shown in the insert of Fig 3B and confirms that almost all cells are constrained to the glass stripes (99±1%; N = 1362). Finally, once adhered to glass stripes, cells moved nearly exclusively along the stripes and rarely moved across the PEG-gel stripes (see S5 Video). Thus, the migration of these developed AX4 cells on the micropatterned substrate with parallel glass and PEG-gel stripes is effectively one-dimensional and can be used as a model to study 1D migration of adherent amoeboid cells. Importantly, the control experiment described in Methods showed that a substrate topography that includes 1.5 *μ*m deep grooves has minimal effect on cell adhesion and migration (S6 Video). Therefore, the observed one-dimensional motion is due to differences in cell adhesion to PEG-gel and glass rather than to topographic cues.

#### Vegetative cells

**Fig 4:**
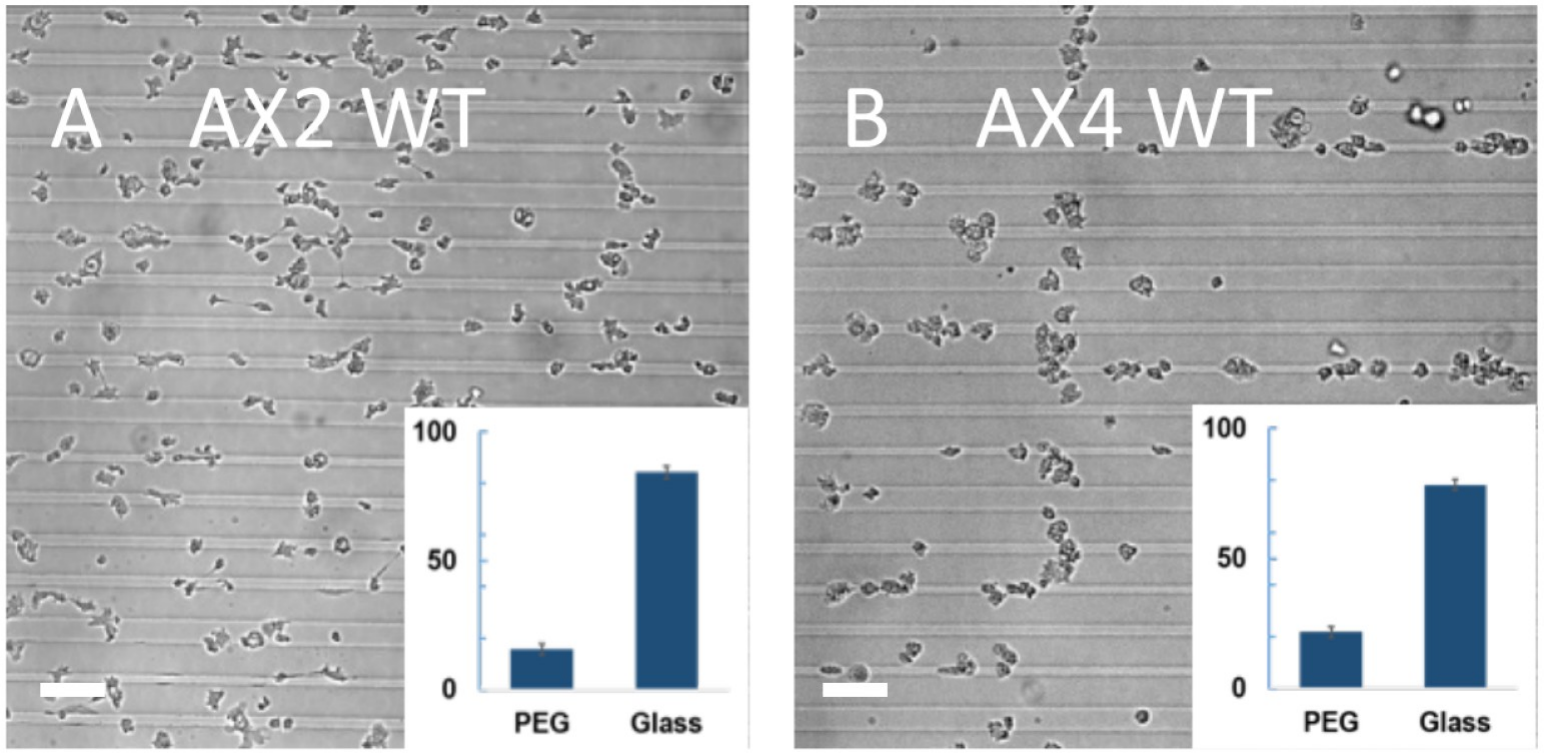
Vegetative cells on micropatterned substrates. A) Micrographs of WT AX2 (A) and AX4 (B) cells, 10 min after plating. Insets: relative coverage of cells on PEG-gel and glass stripes. For both strains, there is a major bias towards the adhesion to glass vs. PEG-gel. (Scale bar: 50 *μm*).

Repeating the above experiments using vegetative AX2 cells we found that only 16±2% of cells were located on the PEG-gel stripes (Fig 4A; N = 457). This is in contrast to developed AX2 cells, which adhere in comparable proportions to both surfaces (cf. Fig 3A). In further contrast with developed cells, vegetative wild-type AX4 cells were found in relatively small clumps, when settled onto the substrate, and these clumps broke up into single cells. As demonstrated in Fig 4B, these cells predominantly adhered to glass stripes, with only 22 ±2% located on PEG-gel stripes (N = 660), and preferentially migrated along glass stripes. Nevertheless, the adhesion to and migration along the glass stripes was not nearly as exclusive as for developed fluorescent AX4 cells (cf. Fig 3B).

### Dependence of adhesion and migration on fluorescent markers

#### Developed cells

**Fig 5:**
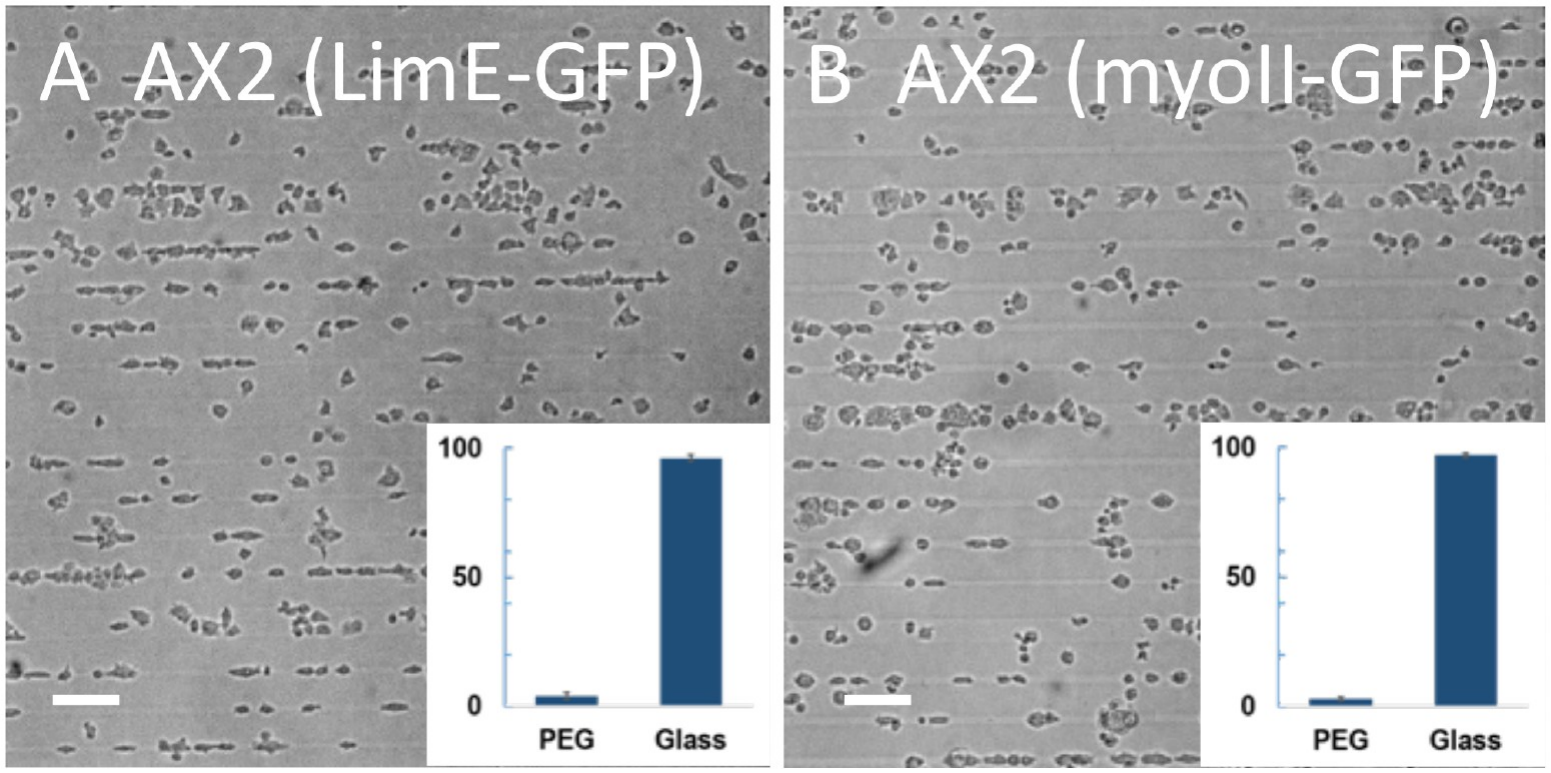
Developed AX2 cells with fluorescent markers on micropatterned substrates. Micrographs of developed AX2 cells expressing LimE-GFP (A) and myoII-GFP (B) on the micropatterned substrate taken 10 min after plating. Insets: relative coverage of cells on PEG-gel and glass stripes. (Scale bar: 50 *μm*).

Our micropatterned substrate results show that developed AX2 cells adhered to glass and PEG-gel stripes in comparable proportions, while developed AX4 cells expressing fluorescent protein markers for LimE and coronin are overwhelmingly constrained to the glass stripes. We have verified that developed AX4 cells that only express LimE are also mainly constrained to the glass stripes (14% on PEG-gel; N = 928; S2 Fig). To test for possible dependence of adhesion and migration on the expression of fluorescent protein markers in developed AX2 cells, we used AX2 cells that expressed either LimE, MyoII, or both. LimE is a label for the cortical actin filament network [34] while MyoII visualizes myosin-II, which interacts with actin to generate contractile forces [35, 36]. The relative coverages of AX2 cells expressing either LimE or MyoII that adhered to PEG-gel stripes were both very small, at 4% (N = 1657) and 3% (N = 1192), respectively (Fig 5A & 5B). In addition, AX2 cells from both fluorescent lines predominantly migrated along the glass stripes. The bias towards the adhesion to glass was even stronger for developed AX2 cells expressing both LimE and MyoII (only 1% of cells on PEG-gel; N = 612; S3A Fig). We should note, however, that the major reduction of the proportion of developed AX2 cells on PEG-gel stripes was not present for every fluorescent marker. For example, for AX2 cells expressing the microtubule marker alpha-tubulin, 39 ± 4% (N = 2456) of cells were found on PEG-gel stripes, and these cells were still able to migrate on the PEG-gel surface (S3B Fig).

#### Vegetative cells

**Fig 6:**
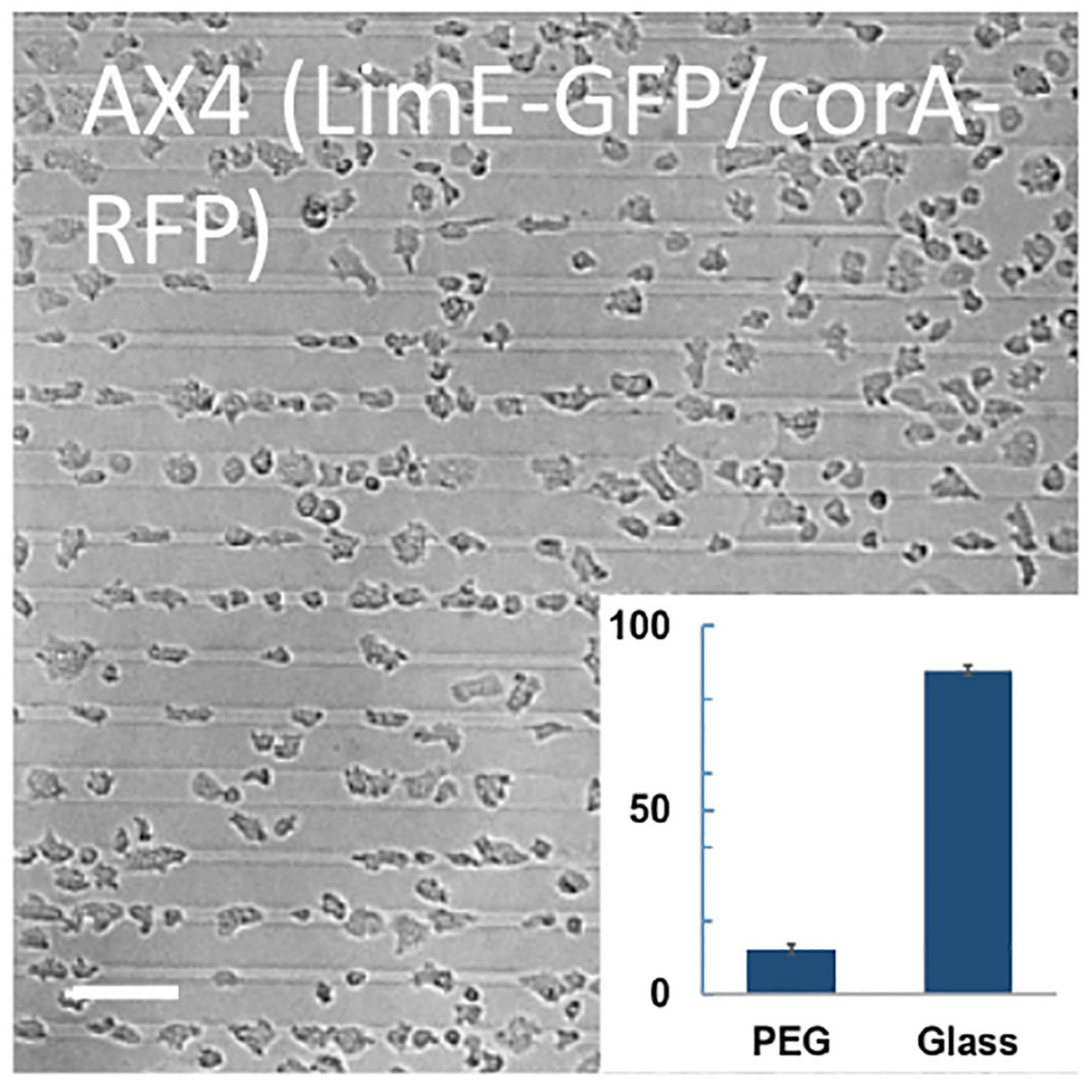
Vegetative AX4 cells with fluorescent markers on micropatterned substrates. Micrograph of vegetative AX4 cells expressing LimE-GFP/corA-RFP taken 10 min after plating. Inset: relative coverage of cells on PEG-gel and glass stripes. (Scale bar: 50 *μm*).

We next examined the ability of vegetative AX4 cells that express markers for LimE and coronin (LimE-GFP/corA-RFP) to adhere to PEG-gel stripes. The results of this experiment are presented in Fig 6 and show that 12 ± 1% (N = 932) of the cells were found on PEG-gel stripes. This was substantially greater as compared with the same cell line in the developed state (Fig 3B) but smaller as compared with vegetative AX4 cells (Fig 4B).

### SCFS measurements of adhesion forces on glass and PEG

The experiments reported above indicate that the differences between the adhesion of *Dictyostelium* cells to glass and PEG-gel surfaces vary between the two axenic strains and degree of development, and can be affected by the expression of fluorescent reporters. To investigate this subject further, we used AFM-based single cell force spectroscopy to measure the forces between a cell and a substrate, as the cell is pulled away from the substrate. As shown in S1 Fig and detailed in Methods, these experiments result in force-distance (FD) curves which can be used to quantify the maximum adhesion force *F*_*max*_ between a cell and the substrate [28]. Although we focus here on *F*_*max*_, a predictor for the differences in adhesion to PEG-gel and glass on the micropatterned substrates, we have also used the FD curves to compute the work of adhesion *W*_*adh*_ and report the results in the Supporting Information. Sample FD curves are presented in S4 Fig.

#### Developed cells

**Fig 7:**
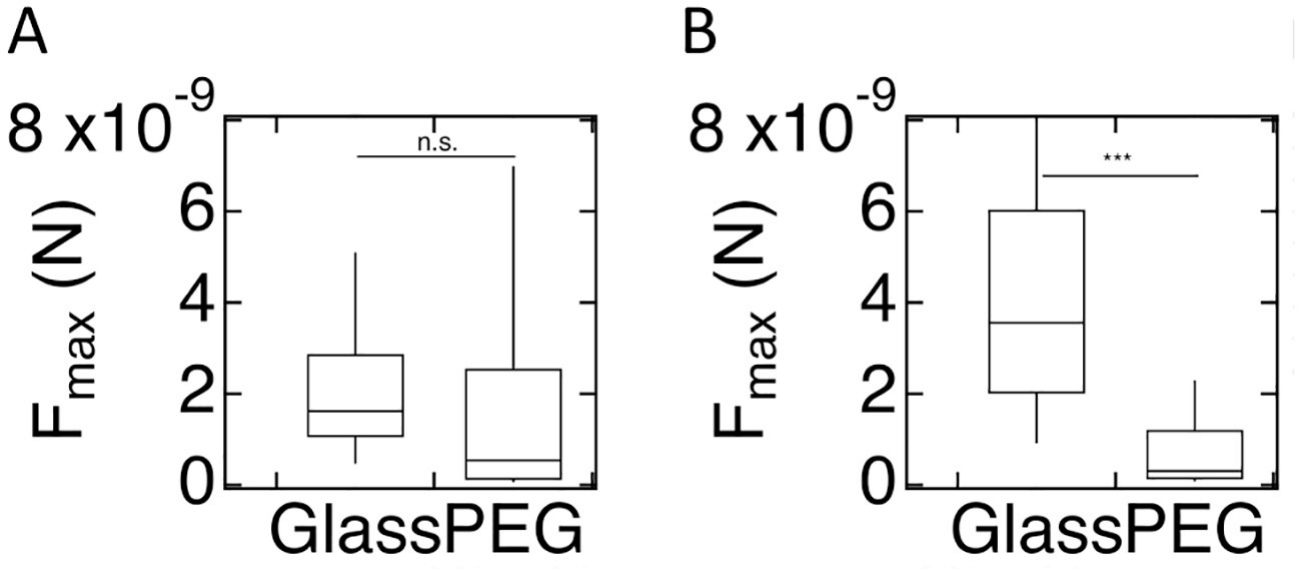
Maximum force of adhesion for developed cells. A) Box plot of the distribution of *F*_*max*_ for WT AX2 cells on glass and PEG-gel surface, where the bottom and the top of the box represents the first and the third quartiles, and the band corresponds to the median. The difference in *F*_*max*_ is not significant. B) As in A, but now for WT AX4 cells. For these cells, the difference in *F*_*max*_ is significant.

Our results for *F*_*max*_ for developed WT AX2 cells are summarized in Fig 7A. The results show that median *F*_*max*_ for these cells displays a statistically non-significant reduction on PEG-gel surfaces as compared to glass surfaces (5 ×10^−10^ (1.3×10^−10^-2.5×10^−9^)N vs. 1.6×10^−9^ (1.1×10^−9^ to 2.8×10^−9^)N; p = 0.48). The number of repeat experiments, cells, and FD curves for these SCFS experiments is reported in S1 Table. In contrast, *F*_*max*_ for developed WT AX4 cells on PEG-gel is significantly reduced as compared to glass (Fig 7B; 3×10^−10^ (1.5×10^−10^-1.2×10^−9^)N vs. 3.6×10^−9^ (2.0×10^−9^-6.0×10^−9^)N; p<0.001). A table detailing the p-values obtained comparing the statistics of the different experiments is presented in S2 Table. Note that these results are consistent with the results of experiments on the adhesion of developed AX2 and AX4 cells to uniform glass and PEG-gel substrates (Fig 2) and to micropatterned substrates (Fig 3). The results for *W*_*adh*_, along with a detailed list of p-values, are presented in S5 Fig and S3 Table.

#### Vegetative cells

**Fig 8:**
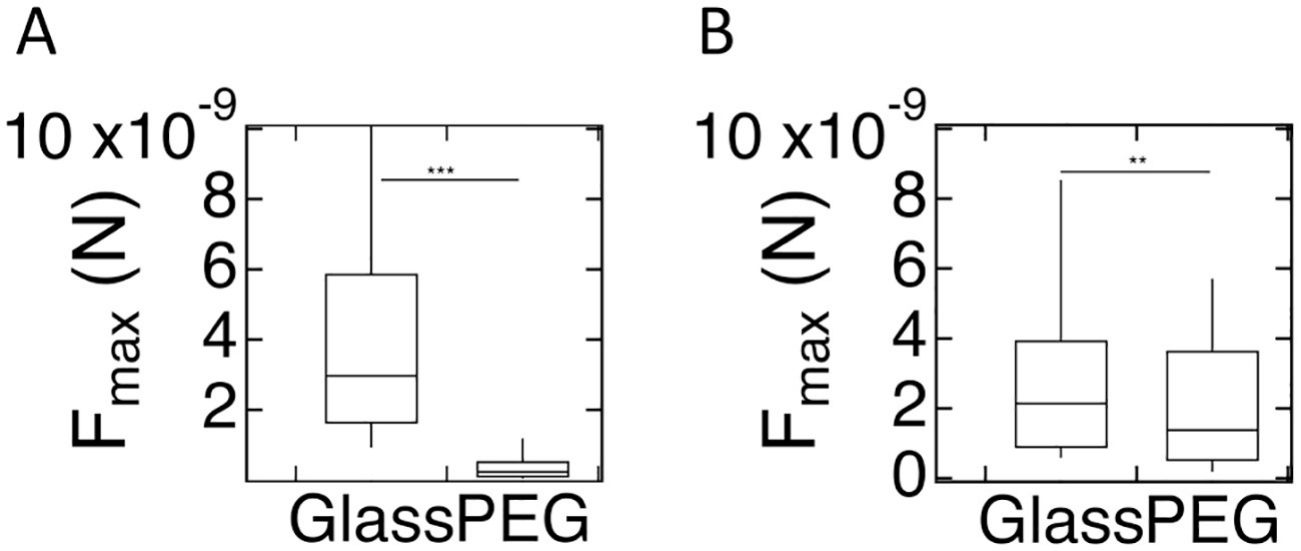
SCFS results for vegetative WT cells. *F*_*max*_ for AX2 (A) and AX4 (B) cells on glass and PEG-gel surfaces.

The results of the SCFS experiments with vegetative WT cells are presented in Fig 8. *F*_*max*_ is significantly reduced for AX2 cells on the PEG-gel as compared with glass surface (2×10^−10^ (1.1×10^−10^-5×10^−10^)N vs. 3.0×10^−9^ (1.6×10^−9^-5.8×10^−9^)N p<0.001). *F*_*max*_ for vegetative AX4 cells is also significantly different for AX4 cells on PEG-gel vs. glass: (1.4×10^−9^ (5×10^−10^-3.6×10^−9^)N vs. 2.1×10^−9^ (9×10^−10^-3.9×10^−9^)N, p = 0.006). Corresponding results for *W*_*adh*_ are shown in S6 Fig. The statistics for these experiments are reported in S4 Table while a detailed list of p-values are provided in S5 and S6 Tables.

### SCFS measurements on cells expressing fluorescent markers

#### Developed cells

**Fig 9:**
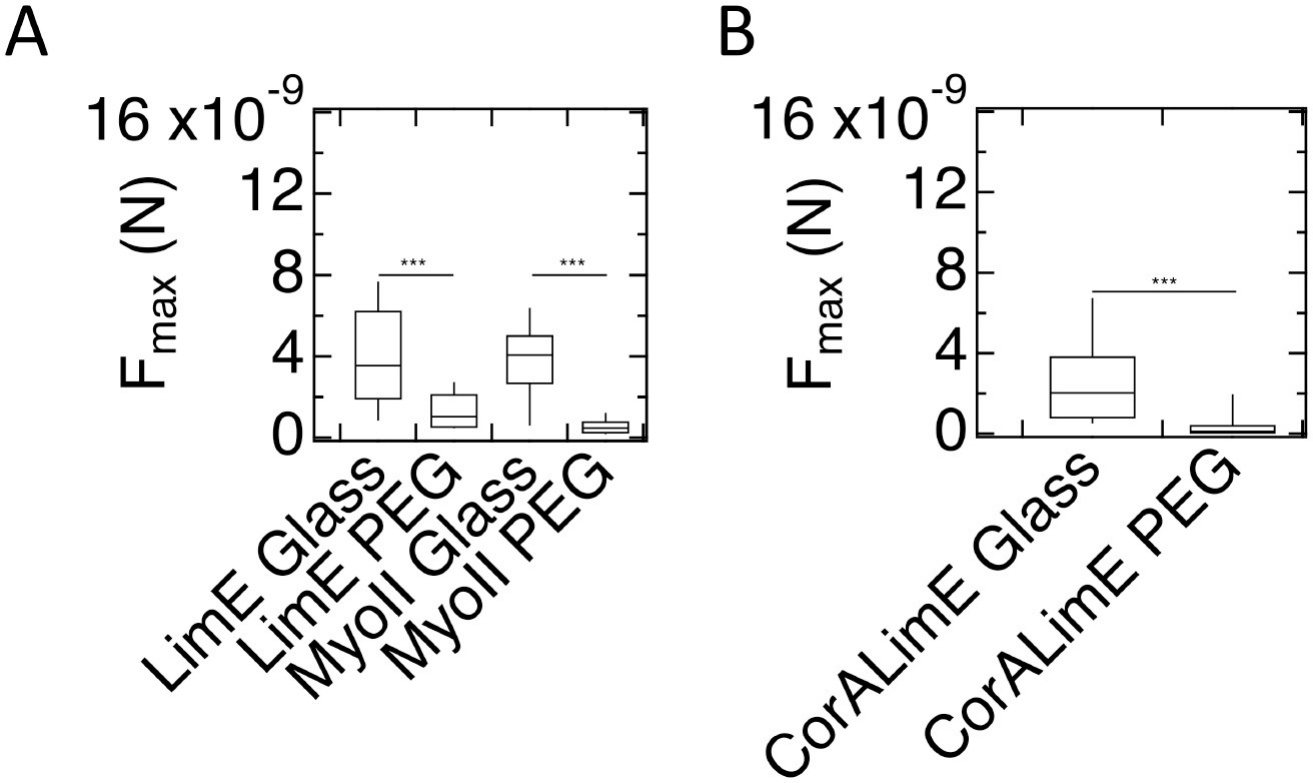
Maximum force of adhesion for developed cells with cytoskeletal labeling. A) AX2 cells with the specified cytoskeletal markers exhibit significantly reduced *F*_*max*_ on PEG-gel surfaces compared to glass. B) As in A), but now for AX4 cells. *F*_*max*_ on PEG-gel surfaces is significantly less than on glass.

We next performed SCFS measurements on cells expressing fluorescent markers of cytoskeletal proteins that were used in the assays on adhesion to and migration on the micropatterned substrate described above (for details on the number of cells and curves, see S7 Table). The results of these experiments are summarized in Fig 9 for AX2 (A) and AX4 cells (B). The values of *F*_*max*_ on PEG-gel were significantly smaller than on glass for both AX2 and AX4 cells expressing cytoskeletal markers (p<0.001 for all markers). This result is consistent with the very small proportions of developed cytoskeletal marker-expressing AX2 cells adhered to PEG-gel stripes on the micropatterned substrate (Fig 5). All p values, comparing different cell types on different and identical substrates, are reported in S8 Table). The values for AX2 cells on PEG and glass are: *F*_*max*_ = 1.0×10^−9^ (5×10^−10^-2.1×10^−9^)N vs. 3.5×10^−9^ (1.9×10^−9^-6.2×10^−9^)N for LimE-GFP and *F*_*max*_ = 5×10^−10^ (2×10^−10^-7×10^−10^)N vs. 4.1×10^−9^ (2.7×10^−9^-5.0×10^−9^)N for myoII-GFP. For AX4 cells, we found *F*_*max*_ = 1.1×10^−10^ (4×10^−11^-4×10^−10^)N vs. 2.0×10^−9^ (8×10^−10^-3.8×10^−9^)N for LimE-GFP/corA-RFP (p<0.001). Corresponding values for *W*_*adh*_ are presented in Fig. S7A & S7B Fig and S9 Table. In Fig. S7C & S7D Fig we also show the SCFS results for developed AX2 cells expressing both LimE-RFP and MyoII-GFP and for developed AX2 cells expressing alpha-tubulin-GFP. *F*_*max*_ was significantly different on glass vs. PEG for both cells (p<0.001 and p<0.01, respectively) while *W*_*adh*_ was only significantly different for the double labeled cells (p = 0.02). Finally, S8 Fig shows SCFS results for developed AX4 cells expressing either LimE-GFP or MyoII-GFP.

#### Vegetative cells

**Fig 10:**
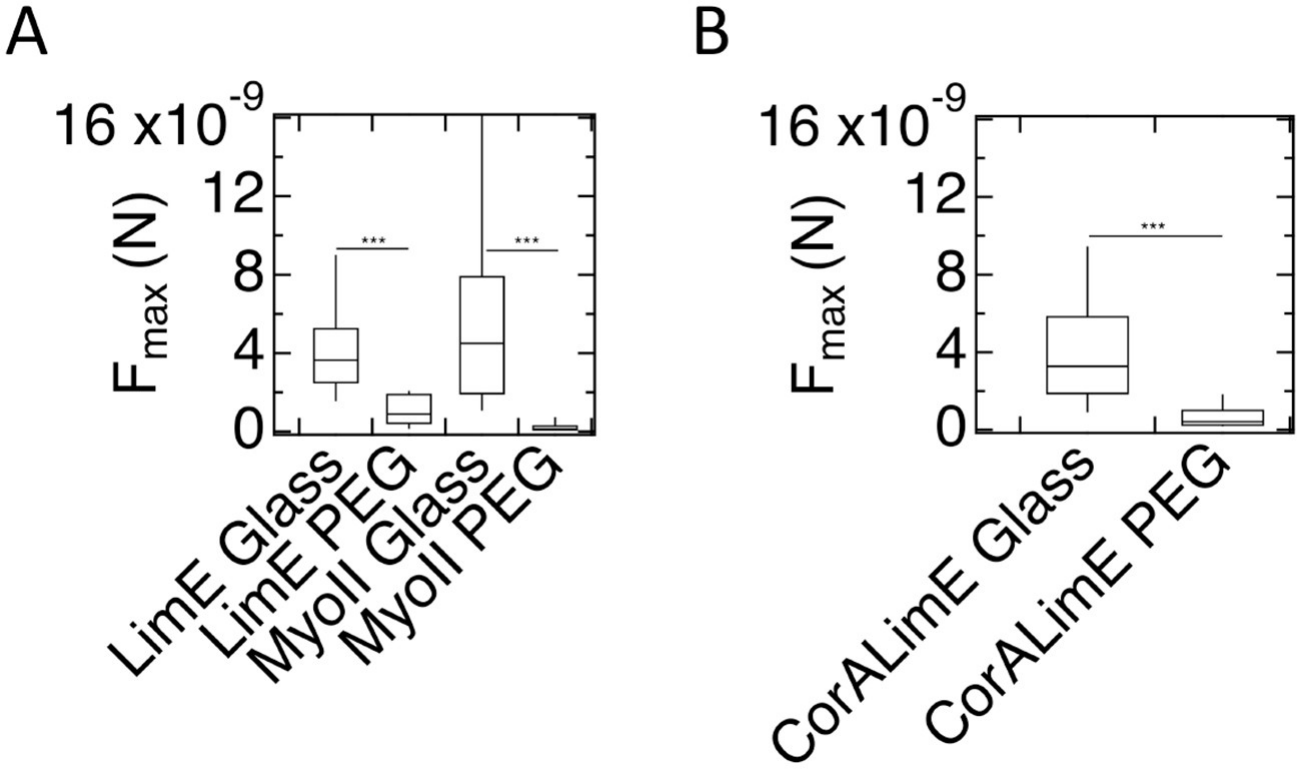
Maximum force of adhesion for vegetative AX2 (A) and AX4 (B) cells with cytoskeletal labeling. *F*_*max*_ on PEG-gel surfaces is significantly less than on glass.

Finally, we performed SCFS measurements on vegetative AX2 and AX4 cells carrying fluorescent cytoskeletal markers (Fig 10), with statistics in S10 Table. For AX2 cells expressing the fluorescent markers LimE and MyoII, *F*_*max*_ is significantly smaller (p<0.001) on PEG-gel than on glass: *F*_*max*_ = 9×10^−10^ (4×10^−10^-1.9×10^−9^)N vs. *F*_*max*_ = 3.6×10^−9^ (2.5×10^−9^-5.9×10^−9^)N for LimE and *F*_*max*_ = 4.5×10^−9^ (1.9×10^−9^-7.9×10^−9^)N vs. *F*_*max*_ = 1×10^−10^ (9×10^−11^-3×10^−9^)N for MyoII. The corresponding *W*_*adh*_ for these cells is presented in Fig. S9A Fig. For AX4 cells that express LimE-GFP/corA-RFP, we find for PEG-gel and glass values of *F*_*max*_ = 4×10^−10^ (2×10^−11^-1.0×10^−9^)N and *F*_*max*_ = 3.3×10^−9^ (1.9×10^−9^-5.8×10^−9^)N, respectively. This difference is greater than for WT AX4 cells (Fig 8). The corresponding *W*_*adh*_ for these cells is shown in S9B Fig. For completeness, we show the SCFS results for vegetative AX4 cells expressing either LimE-GFP or MyoII-GFP in S8 Fig.

## Discussion

The motivation for our work was to develop a relatively simple and reliable experimental system, where the migration of adherent amoeboid *Dictyostelium* cells is largely one-dimensional (1D), along narrow stripes. Unlike adherent mammalian cells that have dedicated adhesion complexes with specialized adhesion proteins, *Dictyostelium* cells employ non-specific van der Waals and electrostatic interactions for their adhesion [21, 22, 28] and adhere to and migrate on a large variety of substrates. In fact, *Dictyostelium* cells are so “sticky” that it is difficult to find a “passive” substrate to which they would not adhere. A widely used method to passivate a substrate in order to block cell adhesion is by coating the substrate with macromolecular “brushes” of PEG. *Dictyostelium* cells were reported to not adhere to a commercial glass substrate coated with a high density of PEG molecules [25]. Our attempts, however, to block the adhesion of *Dictyostelium* cells by coating glass surface with brushes of PEG (e.g., by using various formulations of Pluronic with different pre-treatments of the glass surface) were never completely successful. This may be because the density of PEG was not sufficiently high and cellular processes could penetrate it. On the other hand, we have been able to completely block the adhesion of *Dictyostelium* cells (Fig 2) by coating a glass substrate with an ∼ 1*μ*m layer of a 30% PEG-gel. This dense layer of gel is too thick for any van der Waals and electrostatic interactions between cell and glass and is able to hide the glass substrate from cells on the surface of the gel. We believe that coating a substrate with a thin layer of this PEG-gel is an appealing way to block the adhesion of *Dictyostelium* cells (and other sticky cells), because it is efficient, robust, and reliable, while being relatively simple and easy. Most importantly, PEG-gel coating enables preparation of micropatterned surfaces with sharp edges on a microscope coverglass (compatible with high-resolution inverted microscopes), using a relatively simple laboratory protocol. This protocol only requires a reusable microfluidic perfusion device made of PDMS, a low-intensity UV light derived from an inexpensive 365 nm UV LED, and an *N*_2_ chamber, and uses inexpensive commercial reagents for coverglass treatment and PEG-gel pre-polymer preparation. The proposed protocol is more accessible and less labor intensive than the protocol of micropatterning with PEG brushes described in Ref. [24] that required performing photolithography directly on the substrate (spin-coating the substrate with a photoresist, exposing it to collimated UV-light through a photomask, development the photoresist, etc.) and produced micropatterns on thick glass slides. The small thickness of the PEG-gel layer minimizes the effect of vertical walls of PEG around the glass stripes such that cells are not constrained by these walls [37]. On the other hand, unlike micropatterns of PEG brushes, PEG-gel micropatterns are readily visible under a microscope, which is a clear advantage. In this study, we used our protocol to create micropatterned surfaces with parallel stripes of glass and PEG-gel surface. The most important practical outcome of this micropatterning is that the adhesion and migration of *Dictyostelium* cells is nearly exclusively limited to the glass stripes (Figs 3B and 5), thus creating a model system to study 1D migration of adherent amoeboid cells. As a final note, by coating glass stripes with ECM proteins (that are not likely to adhere to PEG-stripes), it may be possible to adapt the proposed substrates for experiments on adhesion and migration of mammalian cells.

Interestingly, we found that the ability of *Dictyostelium* cells to adhere to PEG-gel surfaces depends in a relatively complex way on several factors. First, it depends on the degree of development of the cells, most notably for WT cells of the parental strain AX4. Developed AX4 cells cannot adhere to PEG-gel surfaces and, when plated on these surfaces, are unable to migrate to a pipette releasing cAMP (Figs 2 and 3, S2 Video). Vegetative cells of the same strain, however, can adhere to PEG-gel, and when these cells are plated on the micropatterned substrate, a substantial proportion of them are found on PEG-gel stripes (Fig 4 and S4 Video). In contrast, the relative coverage of AX2 cells on PEG-gel increases slightly as the cells develop (Figs 3 and 4). Similar development-dependent cell-substrate adhesion changes were already observed in an earlier study with AX3 cells [22]. The reasons for these changes are currently unclear but point towards differences in processes that occur during development. The elucidation of these processes would require further studies.

Second, adhesion of *Dictyostelium* cells to PEG-gel strongly depends on their parental strain. Most strikingly, while developed AX4 cells cannot adhere to PEG-gel surfaces, developed AX2 cells can (Fig 2, S1 and S2 Videos). In fact, the maximum forces of retraction, *F*_*max*_, for AX2 cells from glass and PEG-gel surfaces, as measured with SCFS, are nearly identical (Fig 7). As a result, when plated on the micropatterned substrate, AX2 cells adhere to PEG-gel and glass stripes in comparable proportions (Fig 3A). We should point out that, whereas the difference in *F*_*max*_ between AX2 cells on PEG-gel and glass surfaces was not significant, the difference in the total work of adhesion, *W*_*adh*_, was significant (Fig 1). This result suggests that the difference in *F*_*max*_ may be a better predictor of proportions of cells found on the different types of surfaces, when plated onto a micropatterned substrate.

A number of previous studies have reported strain dependent behavior in *Dictyostelium*. For example, Dormann and Weijer have shown that the presence of optical density waves in slugs, generated by periodic cell movement, is strain dependent. Some strains almost always displayed density waves, while others never displayed them [38]. Strain-dependent differences in spontaneously forming, large scale multi-cellular patterns were also observed when *Dictyostelium* cells were grown in confinement [39]. Strain dependency is also present in the phenotypic behavior of single cells. For example, a careful and exhaustive study of localization of SCAR, a regulator of actin polymerization, and cell motility of vegetative *Dictyostelium* cells found significant differences between strains [40]. These differences may be attributable to different duplications of stretches of the genome in the different *Dictyostelium* strains [41]. Strain-dependent genetic differences have also been reported in the expression of talin [42], a protein that is believed to be involved in cell-substrate adhesion [43]. Specifically, unpublished data reports that AX4 cells have a truncated talin A protein while AX2 talin is the full length homolog of mammalian talin [42]. Importantly, however, AX4 cells are not talin-null mutants. This can be deduced from a recent study, which examined the adhesive properties of cells of the strain AX3, an ancestor of AX4 [22]. The study used both an SCFS assay and a microfluidic device that generated a range of hydrodynamic shear stresses. It showed that mutant AX3 cells that lack talin have significantly different adhesive properties than wild type AX3 cells. In particular, the adhesive properties of the mutant cells are very similar to those of vegetative cells, in sharp contrast with wild-type cells in which adhesion drops 10-fold during the first few hours of development. Thus, the difference in adhesion between our two strains cannot be due to the absence of talin in AX4. Perhaps, as speculated earlier [22], talin affects the rigidity of the membrane since it has been previously shown to couple force generation to cellular morphogenesis [44]. Future studies could focus on characterizing the role of cortical tension and membrane bending moduli with AFM for AX2 and AX4 cells lacking talin in a similar way as it has been done earlier for the talin-null AX2 cells using RICM and on different surface compositions [23, 45].

Third, and perhaps most striking, cell-substrate adhesion appears to be affected by the expression of fluorescent protein markers. Most dramatically, while the difference between adhesion of cells to PEG-gel and glass for developed WT AX2 cells, as measured by *F*_*max*_, is small and statistically insignificant, this difference becomes large and significant when fluorescent markers of actin and myosin are expressed (Fig 9). Consistent with the large difference in *F*_*max*_, when plated on the micropatterned substrate, these fluorescent cells nearly exclusively adhered to glass stripes (Fig 5). This effect is not universal for all fluorescent protein markers or all components of cytoskeleton, because developed AX2 cells expressing a fluorescent marker for tubulin, when plated on the micropatterned substrate, adhered to PEG-gel and glass in nearly equal proportions (S3 Fig).

The fact that we find that expressing or introducing a fluorescent marker in a cell can affect the phenotypic behavior may not be that surprising. After all, this changes the amount of available binding sites or the diffusive behavior of the relevant proteins, which could result in phenotypic changes. This reasoning is consistent with a recent study that showed that Lifeact, a marker for F-actin, can induce dose-response artefacts at the cellular level, most likely due to reduced binding of cofilin to actin filaments [46]. Furthermore, several reports have found that the expression of fluorescent protein can alter cell behavior. For example, it was found that GFP expression in rat muscle cells can impair actin-myosin interactions [47]. In addition, a recent study showed that GFP expression in breast cancer cells can induce changes in expression of proteins that are associated with protein folding, cytoskeletal organisation and cellular immune response [48]. Finally, the expression of GFP-myosin in *Dictyostelium* cells was found to rescue all myosin null cell defects [49]. Our work shows that the presence of fluorescent markers can also result in altered cellular adhesion.

## Summary

The study shows that the adhesion of *Dictyostelium* cells to PEG-gel depends on their parental strain, degree of development, and the expression of fluorescent protein markers. We also show that this finding can be used to prepare micropatterned substrates on which the adhesion of sticky *Dictyostelium* is restricted to narrow stripes of glass. The findings of our study may help interpret results of experiments on cell-substrate adhesion.

## Supporting information

S1 Fig Schematic setup for single cell force spectroscopy (SCFS). SCFS involves repeated cycles of approach and retraction of a cantilever-attached *Dictyostelium* cell (inset, bottom, and scheme top left), resulting in force-distance (FD) curves (top right). These FD curves can be used to determine *F*_*max*_ and *W*_*adh*_. For further details, see Methods.
(PDF)

S2 Fig Micrograph of developed AX4 cells expressing LimE-GFP on the micropatterned substrate taken 10 min after plating. Insets: relative coverage of cells on PEG-gel and glass stripes. (Scale bar: 50 *μ*m).
(PDF)

S3 Fig Micrographs of developed AX2 cells expressing both LimE-RFP and myoII-GFP (A) and alpha-tubulin-GFP (B) on the micropatterned substrate taken 10 min after plating. Insets: relative coverage of cells on PEG-gel and glass stripes. (Scale bar: 50 *μ*m).
(PDF)

S4 Fig Representative Force-Distance (FD) curves from SCFS experiments on AX2 and AX4 *Dictyostelium* cells on glass and PEG-gel surfaces. (PDF)

S5 Fig Work of adhesion *W*_*adh*_ for developed WT AX2 (A) and AX4 cells (B). Median *W*_*adh*_ for PEG-gel is vs. glass 3.6×10^−16^ (6×10^−17^-1.2×10^−15^)J vs. 1.72×10^−15^(2.9×10^−16^-1.27×10^−14^)J for AX2 and 3.6×10^−15^ (7×10^−16^-1.12×10^−14^)J vs. 2.8×10^−16^(6×10^−17^-1.8×10^−15^)J for AX4 cells (p<0.001 for both).
(PDF)

S6 Fig *W*_*adh*_ on glass and PEG-gel surfaces for vegetative AX2 (A; 4.4×10^−16^ (1.0×10^−16^-1.0×10^−15^)J vs. 4.0×10^−15^(5.8×10^−16^-1.22×10^−14^)J and for vegetative AX4 cells (B; 1.3×10^−15^ (2×10^−16^-7.0×10^−15^)J vs. 1.3×10^−15^(2×10^−16^-7.0×10^−15^)J). (PDF)

S7 Fig Additional SCFS data: *W*_*adh*_ for developed AX2 cells expressing LimE-GFP or myoII-GFP (A) and for developed AX4 cells expressing LimE-GFP/corA-RFP (B). *F*_*max*_ for developed AX2 cells expressing both LimE-RFP and myoII-GFP, and alpha-tubulin-GFP (C) and the corresponding *W*_*adh*_ (D).
(PDF)

S8 Fig *F*_*max*_ (A) and *W*_*adh*_ (B) for vegetative and developed fluorescently labeled AX4 cells on glass and PEG. (PDF)

S9 Fig *W*_*adh*_ for vegetative AX2 cells expressing LimE-GFP and MyoII-GFP (A) and AX4 cells expressing LimE-GFP/corA-RFP (B). (PDF)

S1 Video Developed WT AX2 cells on a substrate with a uniform PEG-gel layer. The movie shows the response of WT AX2 cells to a gradient of chemoattractant cAMP leaking from a pipette tip. Cells are able to adhere to the PEG-gel surface, gain traction, and start migrating towards the pipette tip.
(WMV)

S2 Video Developed WT AX4 cells on a substrate with a uniform PEG-gel layer. The movie shows the response of wildtype AX4 cells to a gradient of chemoattractant cAMP leaking from a pipette tip. Cells are unable to adhere to the PEG-gel, appear to float on top of it, and remain in clumps.
(WMV)

S3 Video Vegetative WT AX2 cells on a substrate with a uniform PEG-gel layer. The movie shows that cells are able to adhere to the surface and gain traction.
(WMV)

S4 Video Vegetative WT AX4 cells on a substrate with a uniform PEG-gel layer. Cells are able to adhere to the surface and gain traction.
(WMV)

S5 Video Developed AX4 cells expressing LimE and coronin on a micropatterned substrate. The movie shows that these cells migrate along glass stripes but not PEG-gel stripes.
(AVI)

S6 Video Developed AX4 cells expressing LimE and coronin plated directly on the microfluidic chip. The movie shows that these cells migrate without any topographic guidance.
(AVI)

S1 Table Statistics for SCFS measurement with WT developed cells. Reported here, and in other tables, are the number of separate experiments (N_days_), total number of cells (N_cells_), and the number of FD curves (N_curves_).
(PDF)

S2 Table p-values for *F*_*max*_. Here, and in the other tables, the matrix shows the p values for the distributions on different substrates and for different cell types (see Methods).
(PDF)

S3 Table p-values for *W*_*adh*_. (PDF)

S4 Table Statistics for SCFS measurements using WT vegetative cells. (PDF)

S5 Table p-values for *F*_*max*_. (PDF)

S6 Table p-values for *W*_*adh*_. (PDF)

S7 Table Statistics for SCFS experiments using developed, labeled cells. (PDF)

S8 Table p-values for *F*_*max*_ (developed AX2 cells). (PDF)

S9 Table p-values for *W*_*adh*_ (developed AX2 cells). (PDF)

S10 Table Statistics for SCFS experiments using vegetative, labeled cells. (PDF)
