# Supplementary material for: Novel micropatterning technique reveals dependence of cell-substrate adhesion and migration of social amoebas on parental strain, development, and fluorescent markers
Source: PLoS One. 2020 Jul 23;15(7):e0236171. doi: 10.1371/journal.pone.0236171 (PMC7377449; doi:10.1371/journal.pone.0236171)
Supplement: S1 Fig — SCFS involves repeated cycles of approach and retraction of a cantilever-attached Dictyostelium cell (inset, bottom, and scheme top left), resulting in force-distance (FD) curves (top right). These FD curves can be used to determine Fmax and Wadh. For further details, see Methods. (PDF) [file pone.0236171.s001.pdf]

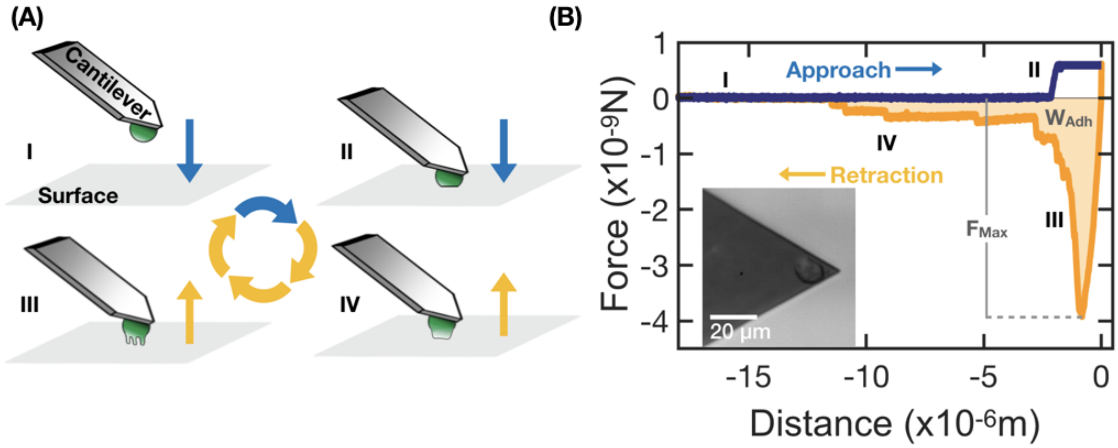

Supporting information

S1 Fig. Schematic setup for Single Cell Force Spectroscopy (SCFS). SCFS involves repeated cycles of approach and retraction of a cantilever-attached *Dictyostelium* cell (inset, bottom, and scheme top left), resulting in force-distance (FD) curves (top right). These FD curves can be used to determine F_{max} and W_{adh}. For further details, see Methods.
