# Supplementary material for: Novel micropatterning technique reveals dependence of cell-substrate adhesion and migration of social amoebas on parental strain, development, and fluorescent markers
Source: PLoS One. 2020 Jul 23;15(7):e0236171. doi: 10.1371/journal.pone.0236171 (PMC7377449; doi:10.1371/journal.pone.0236171)
Supplement: S2 Fig — Insets: relative coverage of cells on PEG-gel and glass stripes. (Scale bar: 50 μm). (PDF) [file pone.0236171.s002.pdf]

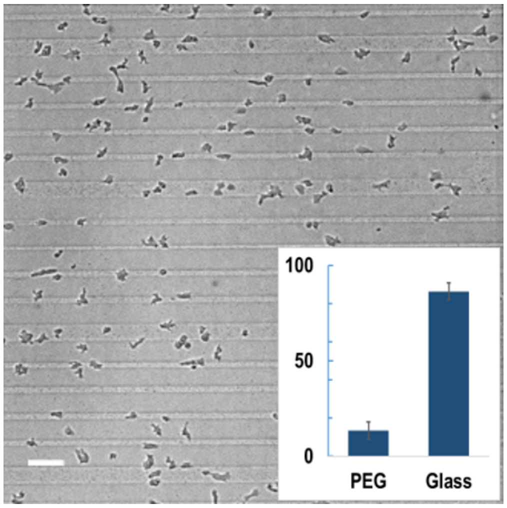

S2 Fig. Micrograph of developed AX4 cells expressing LimE-GFP on the micropatterned substrate taken 10 min after plating. Insets: relative coverage of cells on PEG-gel and glass stripes. (Scale bar: 50 μm)
