# Supplementary material for: Novel micropatterning technique reveals dependence of cell-substrate adhesion and migration of social amoebas on parental strain, development, and fluorescent markers
Source: PLoS One. 2020 Jul 23;15(7):e0236171. doi: 10.1371/journal.pone.0236171 (PMC7377449; doi:10.1371/journal.pone.0236171)
Supplement: S4 Fig — (PDF) [file pone.0236171.s004.pdf]

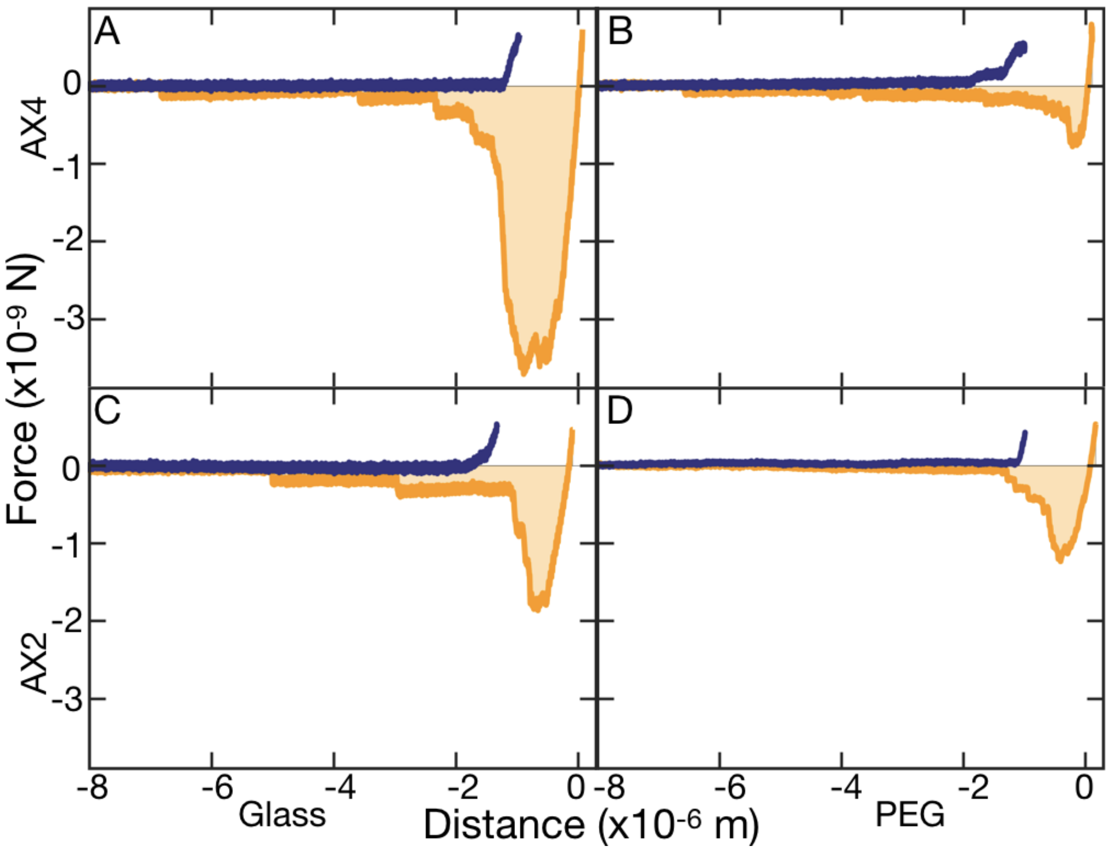

S4 Fig. Representative Force-Distance (FD) curves from SCFS experiments on AX2 and AX4 *Dictyostelium* cells on glass and PEG-gel surfaces.
