# Supplementary material for: Novel micropatterning technique reveals dependence of cell-substrate adhesion and migration of social amoebas on parental strain, development, and fluorescent markers
Source: PLoS One. 2020 Jul 23;15(7):e0236171. doi: 10.1371/journal.pone.0236171 (PMC7377449; doi:10.1371/journal.pone.0236171)
Supplement: S5 Fig — Median Wadh for PEG-gel is vs. glass 3.6×10^−16^ (6×10^−17^-1.2×10^−15^)J vs. 1.72×10^−15^(2.9×10^−16^-1.27×10^−14^)J for AX2 and 3.6×10^−15^ (7×10^−16^-1.12×10^−14^)J vs. 2.8×10^−16^(6×10^−17^-1.8×10^−15^)J for AX4 cells (p<0.001 for both). (PDF) [file pone.0236171.s005.pdf]

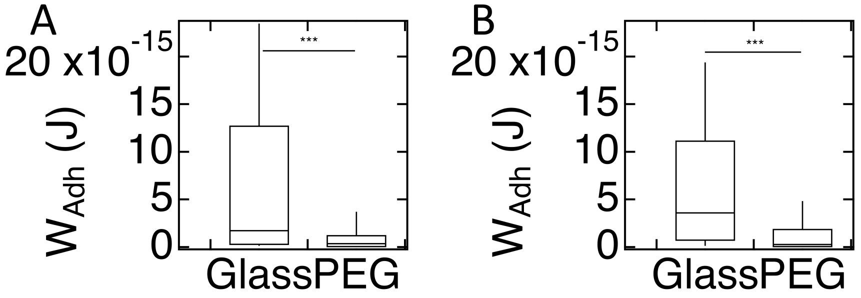

S5 Fig. Work of adhesion W_{adh} for developed WT AX2 (A) and AX4 cells (B). Median W_{adh} for PEG-gel is vs. glass 3.6×10^{-16} (6×10^{-17}-1.2×10^{-15})J vs. 1.72×10^{-15}(2.9×10^{-16}-1.27×10^{-14})J for AX2 and 3.6×10^{-15} (7×10^{-16}-1.12×10^{-14})J vs. 2.8×10^{-16}(6×10^{-17}-1.8×10^{-15})J for AX4 cells ($p<0.001$ for both).
