# Supplementary material for: Novel micropatterning technique reveals dependence of cell-substrate adhesion and migration of social amoebas on parental strain, development, and fluorescent markers
Source: PLoS One. 2020 Jul 23;15(7):e0236171. doi: 10.1371/journal.pone.0236171 (PMC7377449; doi:10.1371/journal.pone.0236171)
Supplement: S6 Fig — (PDF) [file pone.0236171.s006.pdf]

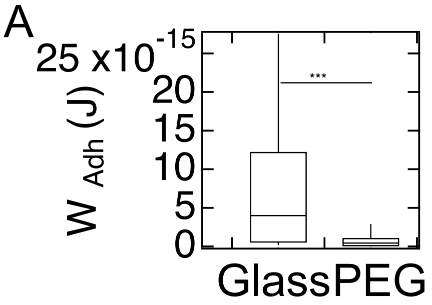

S6 Fig. W_{adh} on glass and PEG-gel surfaces for vegetative AX2 (A; $4.4{\times}10^{-16}$ ($1.0{\times}10^{-16}$-$1.0{\times}10^{-15}$)J vs. $4.0{\times}10^{-15}$($5.8{\times}10^{-16}$-$1.22{\times}10^{-14}$)J and for vegetative AX4 cells (B; $1.3{\times}10^{-15}$ ($2{\times}10^{-16}$-$7.0{\times}10^{-15}$)J vs. $1.3{\times}10^{-15}$($2{\times}10^{-16}$-$7.0{\times}10^{-15}$)J).
