# Supplementary material for: Novel micropatterning technique reveals dependence of cell-substrate adhesion and migration of social amoebas on parental strain, development, and fluorescent markers
Source: PLoS One. 2020 Jul 23;15(7):e0236171. doi: 10.1371/journal.pone.0236171 (PMC7377449; doi:10.1371/journal.pone.0236171)
Supplement: S7 Fig — Fmax for developed AX2 cells expressing both LimE-RFP and myoII-GFP, and alpha-tubulin-GFP (C) and the corresponding Wadh (D). (PDF) [file pone.0236171.s007.pdf]

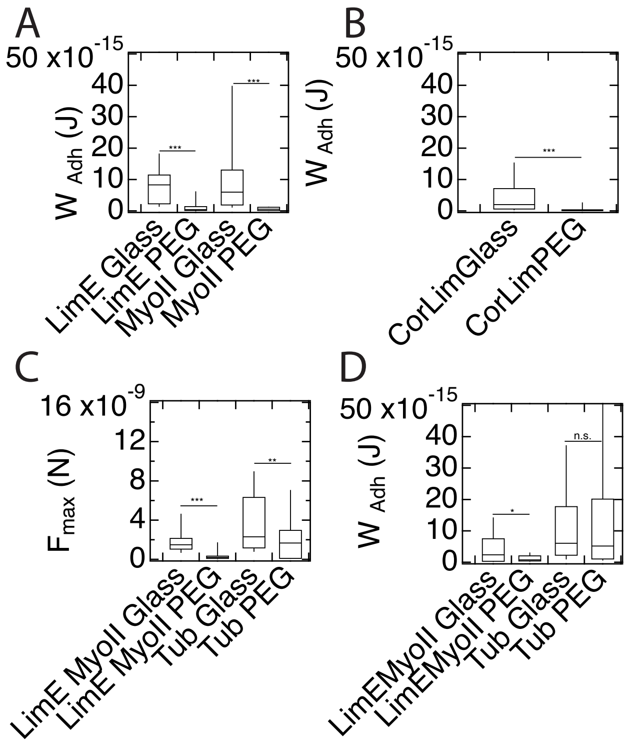

S7 Fig. Additional SCFS data: W_{adh} for developed AX2 cells expressing LimE-GFP or myoII-GFP (A) and for developed AX4 cells expressing LimE-GFP/corA-RFP (B). F_{max} for developed AX2 cells expressing both LimE-RFP and myoII-GFP, and alpha-tubulin-GFP (C) and the corresponding W_{adh} (D).
