# Supplementary material for: Novel micropatterning technique reveals dependence of cell-substrate adhesion and migration of social amoebas on parental strain, development, and fluorescent markers
Source: PLoS One. 2020 Jul 23;15(7):e0236171. doi: 10.1371/journal.pone.0236171 (PMC7377449; doi:10.1371/journal.pone.0236171)
Supplement: S8 Fig — (PDF) [file pone.0236171.s008.pdf]

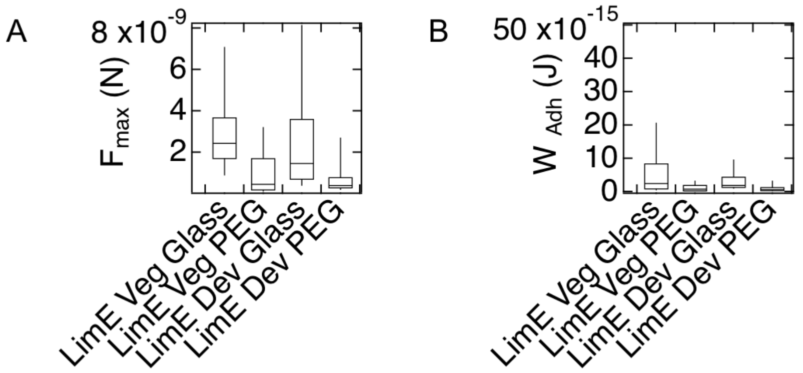

S8 Fig. F_{max} (A) and W_{adh} (B) for vegetative and developed fluorescently labeled AX4 cells on glass and PEG.
