# Supplementary material for: Novel micropatterning technique reveals dependence of cell-substrate adhesion and migration of social amoebas on parental strain, development, and fluorescent markers
Source: PLoS One. 2020 Jul 23;15(7):e0236171. doi: 10.1371/journal.pone.0236171 (PMC7377449; doi:10.1371/journal.pone.0236171)
Supplement: S1 Table — Reported here, and in other tables, are the number of separate experiments (Ndays), total number of cells (Ncells), and the number of FD curves (Ncurves). (PDF) [file pone.0236171.s016.pdf]

S1 Table. Statistics for SCFS measurement with WT developed cells. Reported here, and in other tables, are the number of separate experiments (N_{days}), total number of cells (N_{cells}), and the number of FD curves (N_{curves}).

	AX2/Glass	AX2/PEG	AX4/Glass	AX4/PEG
N_{days}	3	4	3	4
N_{cells}	27	18	26	23
N_{curves}	102	113	142	98
