# Supplementary material for: Novel micropatterning technique reveals dependence of cell-substrate adhesion and migration of social amoebas on parental strain, development, and fluorescent markers
Source: PLoS One. 2020 Jul 23;15(7):e0236171. doi: 10.1371/journal.pone.0236171 (PMC7377449; doi:10.1371/journal.pone.0236171)
Supplement: S2 Table — Here, and in the other tables, the matrix shows the p values for the distributions on different substrates and for different cell types (see Methods). (PDF) [file pone.0236171.s017.pdf]

S2 Table. p-values for F_{max}. Here, and in the other tables, the matrix shows the p values for the distributions on different substrates and for different cell types (see Methods).

	AX2/Glass	AX2/PEG	AX4/Glass	AX4/PEG
AX2/Glass	-	0.48	< 0.001	< 0.001
AX2/PEG	-	-	< 0.001	0.052
AX4/Glass	-	-	-	< 0.001
