# Supplementary material for: Novel micropatterning technique reveals dependence of cell-substrate adhesion and migration of social amoebas on parental strain, development, and fluorescent markers
Source: PLoS One. 2020 Jul 23;15(7):e0236171. doi: 10.1371/journal.pone.0236171 (PMC7377449; doi:10.1371/journal.pone.0236171)
Supplement: S4 Table — (PDF) [file pone.0236171.s019.pdf]

S4 Table. Statistics for SCFS measurements using WT vegetative cells.

	AX2/Glass	AX2/PEG	AX4/Glass	AX4/PEG
N_{days}	4	3	3	3
N_{cells}	20	24	21	20
N_{curves}	108	116	90	97
