# Supplementary material for: Novel micropatterning technique reveals dependence of cell-substrate adhesion and migration of social amoebas on parental strain, development, and fluorescent markers
Source: PLoS One. 2020 Jul 23;15(7):e0236171. doi: 10.1371/journal.pone.0236171 (PMC7377449; doi:10.1371/journal.pone.0236171)
Supplement: S5 Table — (PDF) [file pone.0236171.s020.pdf]

**S5 Table. p-values for $F_{max}$.**

| | AX2/Glass | AX2/PEG | AX4/Glass | AX4/PEG |
|---|---|---|---|---|
| AX2/Glass | - | $< 0.001$ | 0.007 | $< 0.001$ |
| AX2/PEG | - | - | $< 0.001$ | $< 0.001$ |
| AX4/Glass | - | - | - | 0.006 |
