# Supplementary material for: Novel micropatterning technique reveals dependence of cell-substrate adhesion and migration of social amoebas on parental strain, development, and fluorescent markers
Source: PLoS One. 2020 Jul 23;15(7):e0236171. doi: 10.1371/journal.pone.0236171 (PMC7377449; doi:10.1371/journal.pone.0236171)
Supplement: S7 Table — (PDF) [file pone.0236171.s022.pdf]

S7 Table. Statistics for SCFS experiments using developed, labeled cells.

	AX2 LimE Glass	AX2 Myo Glass	AX4 LimE-corA Glass	AX2 LimE PEG	AX2 Myo PEG	AX4 LimE-corA PEG
N_{cells}	7	14	12	5	6	5
N_{curves}	23	67	59	15	19	13
