# Supplementary material for: Novel micropatterning technique reveals dependence of cell-substrate adhesion and migration of social amoebas on parental strain, development, and fluorescent markers
Source: PLoS One. 2020 Jul 23;15(7):e0236171. doi: 10.1371/journal.pone.0236171 (PMC7377449; doi:10.1371/journal.pone.0236171)
Supplement: S8 Table — (PDF) [file pone.0236171.s023.pdf]

**S8 Table. p-values for $F_{max}$ (developed AX2 cells).**

| | LimE Glass | Myo Glass | LimE PEG | Myo PEG |
|---|---|---|---|---|
| LimE/Glass | - | 0.38 | 0.002 | $< 0.001$ |
| Myo/Glass | - | - | $< 0.001$ | $< 0.001$ |
| LimE/PEG | - | - | - | 0.007 |
