# Supplementary material for: Novel micropatterning technique reveals dependence of cell-substrate adhesion and migration of social amoebas on parental strain, development, and fluorescent markers
Source: PLoS One. 2020 Jul 23;15(7):e0236171. doi: 10.1371/journal.pone.0236171 (PMC7377449; doi:10.1371/journal.pone.0236171)
Supplement: S9 Table — (PDF) [file pone.0236171.s024.pdf]

S9 Table. p-values for W_{adh} (developed AX2 cells).

	LimE Glass	Myo Glass	LimE PEG	Myo PEG
LimE/Glass	-	0.40	< 0.001	< 0.001
Myo/Glass	-	-	< 0.001	< 0.001
LimE/PEG	-	-	-	0.41
